# Intralobar pulmonary sequestration expanding toward the contralateral thorax: two case reports

**DOI:** 10.1186/s12893-017-0313-z

**Published:** 2017-11-28

**Authors:** Hizuru Amano, Jun Fujishiro, Akinari Hinoki, Hiroo Uchida

**Affiliations:** 1 Department of Pediatric Surgery, Saitama Children’s Medical Center, 1-2 Shintoshin, Chuo-ku, Saitama city, Saitama, 330-8777 Japan; 2 Department of Pediatric Surgery, Graduate School of Medicine, The University of Tokyo, 7-3-1 Hongo, Bunkyo-ku, Tokyo, 113-0033 Japan; 3 Department of Pediatric Surgery, Nagoya University Graduate School of Medicine, 65 Tsurumai-cho, Showa-ku, Nagoya, Aichi, 466-8550 Japan

**Keywords:** Intralobar pulmonary sequestration, Thoracoscopic surgery, Infant

## Abstract

**Background:**

Intralobar pulmonary sequestration (ILS) is defined as a portion of parenchyma that is contained within the normal pleural investment of the lung but not connected to the tracheobronchial tree, and supplied by anomalous systemic arteries. As ILS is enveloped within the lobe of the normal lung, it is extremely rare for ILS to invade into the mediastinum. We report two atypical cases of infants with ILS expanded toward the posterior mediastinum and contralateral thorax through the pulmonary ligament.

**Case presentation:**

The first case involved a baby boy diagnosed at 30 weeks gestation with a cystic area in his right lower lobe. A chest computed tomography (CT) scan at 29 days of life showed low-density masses in the right lower lung and posterior mediastinum. A complete thoracoscopic right lower lobectomy was performed at 19 months of age. After ligation of the aberrant systemic artery, the mediastinal mass was pulled into the right pleural cavity. The mass was observed to connect to the right lower lobe mass as a segment of lung parenchyma situated within the normal pleural investment of the lung, and the patient was diagnosed with ILS. The second case involved the detection by chest CT of a left lower lung cystic mass that protruded into the posterior mediastinum and contralateral chest of a one-month-old baby girl. A complete thoracoscopic left lower lobectomy was performed at the age of 18 months, and the cystic mass located in the right thoracic cavity was pulled easily into the left pleural cavity and resected. An anomalous systemic artery was identified and ligated, and the patient was also diagnosed with ILS.

**Conclusions:**

As the pulmonary ligament consists of two layers of mediastinal pleura, lower lung ILS with its visceral pleura covering can, though rarely, protrude into the mediastinum through the pulmonary ligament. Our two extremely rare cases of infants with ILS expanded toward the posterior mediastinum and contralateral thorax were successfully treated using a unilateral thoracoscopic approach. Pre-surgical differential diagnosis of mediastinal masses using contrast-enhanced multiple detector CT is important in informing the appropriate surgical approach.

## Background

Pulmonary sequestration is a rare anomaly defined as an aberrant lung tissue mass that does not communicate with the tracheobronchial tree and receives its blood supply from anomalous systemic arteries [1]. Based on its anatomic relation to the normal lung parenchyma, sequestration is classified as either intralobar pulmonary sequestration (ILS) or extralobar pulmonary sequestration (ELS). Specifically, ILS is embedded in a normal lung with which it shares the same visceral pleura, while ELS lies outside normal lung tissue with its own independent visceral pleura. As ILS consists of a portion of normal lung tissue, it is rarely reported to expand into the mediastinum and further to the opposite side of the chest. Indeed, there has been only one previous report of mediastinal herniation associated with ILS [2] to date. Herein, we report two extremely rare cases of infants with ILS expanded toward the posterior mediastinum and contralateral thorax.

## Case presentation

### Patient 1

**Fig. 1:**
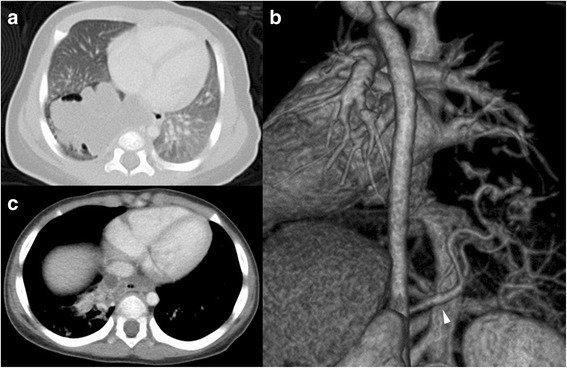
A chest computed tomography scan obtained from Patient 1 at 29 days of life. **a** Diffuse miliary nodules and low-density masses are demonstrated in the right lower lung and posterior mediastinum. **b** An aberrant arterial supply arising from the proximal celiac axis is indicated (arrowhead). **c** The masses appear to be connected to each other through the pulmonary ligament

A baby boy was diagnosed at 30 weeks gestation with a cystic area in his right lower lobe that remained a constant size in utero. He was born at full term and was asymptomatic. His chest radiograph at birth showed consolidation in a portion of the right lower lung. No other congenital anomalies were noted on postnatal physical examination. A chest computed tomography (CT) scan at 29 days of life showed diffuse miliary nodules and low-density masses in the right lower lung and posterior mediastinum (Fig. 1a). The area of the lesion received its blood supply from an anomalous systemic artery originating from the proximal celiac axis (Fig. 1b). Venous drainage returned through the right pulmonary vein and azygos vein. Together, these findings suggested that this right lower lobe mass was consistent with ILS. In contrast, the mass in the mediastinum was thought to be an extrapulmonary lesion; however, the two masses seemed to be connected to each other through the pulmonary ligament (Fig. 1c).

Because the patient was asymptomatic, we chose to delay surgery until he was older. During this time, the lesion spontaneously regressed to some extent. A complete thoracoscopic right lower lobectomy was subsequently performed at 19 months of age after recurring upper respiratory inflammation had subsided. Upon operating, we found that the lung strongly adhered to the diaphragm. After adhesiotomy of the diaphragm from the lower lobe and ligation of the aberrant systemic artery traversing the pulmonary ligament, the mediastinal mass was pulled into the right pleural cavity. This mass was observed to connect to the right lower lobe mass as a segment of lung parenchyma situated within the normal pleural investment of the lung, and was dissected from the esophagus and the mediastinum with little difficulty.

**Fig. 2:**
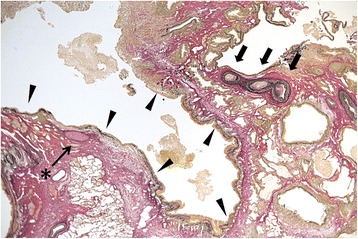
Pathologic analysis of tissue from Patient 1 revealed an elastic artery (aberrant artery) (arrows) and a dilated bronchus (arrowheads) with the presence of cartilage (*) (elastica-van Gieson stain, original magnification ×2)

These operative findings confirmed an operative diagnosis of congenital ILS in the right lower lobe protruding toward the mediastinum through the pulmonary ligament. Pathologic analysis revealed an elastic artery (aberrant artery) and a dilated bronchus with the presence of cartilag, which is also consistent with pulmonary sequestration (Fig. 2). The patient’s postoperative course was uneventful and 7 years after surgery, he is doing well.

### Patient 2

**Fig. 3:**
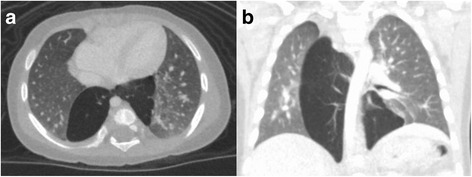
A chest computed tomography scan obtained from Patient 2 at the age of one month. A cystic mass in the left lower lung can be seen protruding into the posterior mediastinum and the opposite side of the chest (**a.** axial, **b.** coronal)

A baby girl was born at 41 weeks gestation. No abnormalities were detected during fetal life, but bilious vomiting and a heart murmur were identified after birth. She was subsequently diagnosed with jejunal atresia and Fallot’s tetralogy, with jejunal atresia being repaired on the fourth day of life. A chest CT scan for follow-up of Fallot’s tetralogy at the age of one month revealed a cystic mass in the left lower lung that protruded into the posterior mediastinum and the opposite side of the chest (Fig. 3 a, b).

As she had no respiratory distress or pulmonary infection, intracardiac repair of Fallot’s tetralogy was performed at the age of two months, while it was decided that surgery for the thoracic mass was to be delayed until the patient was older. The mass did not change in size during this time. A complete thoracoscopic left lower lobectomy was performed at the age of 18 months, where it was found that most of the cystic lesion had expanded toward the posterior mediastinum and the right thoracic cavity through the pulmonary ligament. As there were no adhesions, the cystic mass in the right thoracic cavity was pulled easily into the left pleural cavity. There was no contralateral pneumothorax as the right and left pleural cavities did not communicate. An anomalous systemic artery was also identified and ligated.

**Fig. 4:**
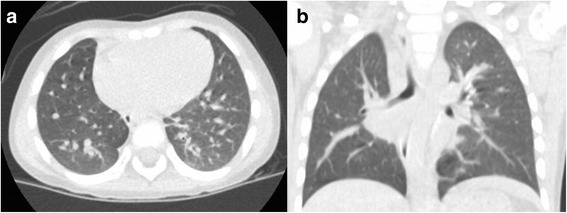
One-year postoperative computed tomography scan obtained from Patient 2. Lungs were well expanded with no residual cystic mass (**a.** axial, **b.** coronal)

These operative findings confirmed an operative diagnosis of ILS expanded toward the posterior mediastinum and contralateral thorax. Pathologic analysis revealed an elastic artery (aberrant artery) and a dilated bronchus with the presence of cartilag, which is also consistent with pulmonary sequestration. The patient’s postoperative course was uneventful and one year after surgery, she is doing well. Her one-year postoperative CT scan is shown in Fig. 4 and demonstrates well expanded lungs with no residual cystic mass.

### Discussion and conclusions

In our cases, the lower lung masses were connected to the mediastinum and protruded further into the opposite side of the chest. Though the masses themselves were technically located in the mediastinum and contralateral thorax, they were found to be ILSs that had expanded from the lower lobes. As ILS is defined as being enveloped within the lobe of the normal lung, ILS protruding into the posterior mediastinum is extremely rare. To the best of our knowledge, only one case of mediastinal herniation associated with ILS has been reported by Fujisawa et al. [2]. In that report, the site through which the mass protruded into the mediastinum was not described. However, from the authors’ CT imaging and operative findings, it is possible that as in our cases, the mass might have expanded toward the contralateral thorax through the pulmonary ligament. As the pulmonary ligament consists of two layers of mediastinal pleura, the lower lung with its visceral pleura covering can protrude into the mediastinum between these two layers; in other words, through the pulmonary ligament. For this reason, it was not difficult in Patient 1 to dissect the masses from the esophagus and mediastinum. With the further expansion of ILS into the contralateral thorax in Patient 2, the expanded lung was easily removed via a unilateral thoracoscopic approach without contralateral pneumothorax.

Cases of mediastinal herniation, such as ours, have been described by Figa et al. [3] in terms of pleural anatomy. Specifically, mediastinal herniation is a condition where there are four pleural layers between the crossover lung tissue and the contralateral lung, and where both pleural cavities do not communicate. In contrast, a horseshoe lung, which is a rare congenital anomaly frequently associated with scimitar syndrome and/or right-lung hypoplasia, is characterized by fusion of the posterobasal segment of both lungs through a partial parietal pleural defect [3]. Although mediastinal herniation and horseshoe lung have similar radiological presentations, with plain radiographs and CT scans showing similarly connected lung masses in both lung segments, it is important to differentially diagnose these conditions based on pleural anatomy. This is because they have different clinical histories and cardiopulmonary anatomy, especially following cardiopulmonary anomalies, such as the scimitar syndrome. Crucially for surgeons, the appropriate surgical approaches for these conditions also occasionally differ. In cases of horseshoe lung, bilateral approaches may be necessary for complete resection, while the protruding lung mass can be resected via a unilateral approach in cases of mediastinal herniation such as ours. Since contrast-enhanced multiple detector CT can reveal vascular, bronchial, and pleural structures, Fujisawa et al. [2], who reported mediastinal lung herniation associated with ILS, recommended it be used as a diagnostic modality for distinguishing between horseshoe lung and mediastinal herniation. The use of this imaging modality to assist with pre-surgical differential diagnosis of masses expanded into the posterior mediastinum should therefore be considered.

In conclusion, as the pulmonary ligament consists of two layers of mediastinal pleura, lower lung ILS with its visceral pleura covering can, though rarely, protrude into the mediastinum through the pulmonary ligament. Our two extremely rare cases of infants with ILS expanded toward the posterior mediastinum and contralateral thorax were successfully treated using a unilateral thoracoscopic approach without contralateral pneumothorax. Pre-surgical differential diagnosis of masses expanded into the posterior mediastinum using contrast-enhanced multiple detector CT is important as different surgical approaches are required for the complete resection of these masses. These atypical presentations emphasize the broad clinical, morphologic, and operative spectra of ILS.

## Abbreviations

CT: Computed tomography
ELS: Extralobar pulmonary sequestration
ILS: Intralobar pulmonary sequestration
